# Visfatin promotes cell and tumor growth by upregulating Notch1 in breast cancer

**DOI:** 10.18632/oncotarget.2086

**Published:** 2014-06-10

**Authors:** Hyun-Joo Park, Su-Ryun Kim, Su Seong Kim, Hee-Jun Wee, Moon-Kyoung Bae, Mi Heon Ryu, Soo-Kyung Bae

**Affiliations:** ^1^ Department of Dental Pharmacology, School of Dentistry, Pusan National University, Yangsan, South Korea; ^2^ Department of Biochemistry, College of Pharmacy, Seoul National University, Seoul, South Korea; ^3^ Department of Oral Physiology, School of Dentistry, Pusan National University, Yangsan, South Korea; ^4^ Department of Oral Pathology, School of Dentistry, Pusan National University, Yangsan, South Korea

**Keywords:** Visfain, Notch1, NF- κB, breast cancer cells

## Abstract

Overexpression of Notch1 has been associated with breast cancer. We recently showed that visfatin stimulates breast cancer cell proliferation and invasion. The present study was undertaken to determine whether Notch1 signaling is affected by visfatin and to characterize the functional role of the visfatin-Notch1 axis in breast cancer. Visfatin and Notch1 were expressed at higher levels in breast tumors than in matched control tissues. Visfatin induced Notch1 expression in MDA-MB-231 breast cancer cell line and in nontransformed MCF10A mammary epithelial cells, whereas visfatin depletion reduced Notch1 mRNA and protein levels. Depletion of Notch1 in MDA-MB-231 cells attenuated cell growth in vitro and in vivo; visfatin depletion produced similar effects, but was less potent. Additionally, Notch1 depletion inhibited cell proliferation induced by visfatin. Analysis of the signaling pathways underlying visfatin-mediated Notch1 upregulation revealed that visfatin activated NF-κB p65. Blockade of NF-κB signaling suppressed the effects of visfatin on Notch1 upregulation and breast cancer cell proliferation. Breast tumors expressing high levels of NF-κB p65 exhibited increased expression of Notch1. Our results demonstrate that the visfatin-Notch1 axis contributes to breast tumor growth through the activation of the NF-κB pathway. Study of the visfatin-Notch1 axis may offer new therapeutic directions for breast cancer.

## INTRODUCTION

Breast cancer is the most common cancers among women worldwide [1]. Recent studies indicate that obesity is a significant risk factor for breast cancer [2]. The influences of obesity and increased adiposity on the risk of breast cancer are partially explained by the changes in adipokines secreted from adipose tissue and from the epithelial tissue of breast tumors [3]. Several adipokines, including leptin [3-5], resistin [3, 6], and hepatocyte growth factor [3, 7], have been associated with an increased risk of breast cancer.

The adipokine visfatin, also known as pre-B-cell colony-enhancing factor [8], is predominantly secreted [8], but it also localizes to the nucleus and cytosol [9]. Visfatin plays roles in inflammation [10] and angiogenesis [11, 12], as well as in circadian rhythms [13], energy metabolism [14], and cell longevity [15] as a nicotinamide mononucleotide adenylyltransferase. Visfatin is associated with a number of human malignancies, including colon, stomach, brain, pancreas, liver, prostate, and breast cancers [16]. Visfatin is highly expressed in human breast cancer cells both *in vitro* and *in vivo* [17-19], and it increases the proliferation and DNA synthesis rate of human breast cancer cells [20], suggesting that visfatin may contribute to breast cancer growth.

Notch family members (Notch1 to Notch4) are large, single-pass type I transmembrane receptors [21]. They are activated by regulated intramembrane proteolysis after interaction with Notch ligands (Delta or Jagged family members) expressed on neighboring cells [21]. Notch signaling has been implicated in a variety of cellular events, including cell fate determination, growth, survival, and differentiation during embryonic and postnatal development [22]. A number of studies implicate Notch dysregulation in the pathogenesis of several human diseases and cancer [23]. Aberrant Notch signaling is involved in breast tumorigenesis: Notch-2 may act as a breast tumor suppressor, whereas Notch1, Notch-3, and Notch4 may act as breast oncogenes [24].

We recently reported that visfatin promotes endothelial angiogenesis through the activation of Notch1 signaling in endothelial cells. However, little information on visfatin-Notch1 interactions in cancer is available. In this study, we show that Notch1 is a downstream target gene of visfatin signaling and describe the role of the visfatin-Notch1 axis in breast cancer cells.

## RESULTS

### Upregulation of visfatin and Notch1 in human breast tumor samples

**Figure 1:**
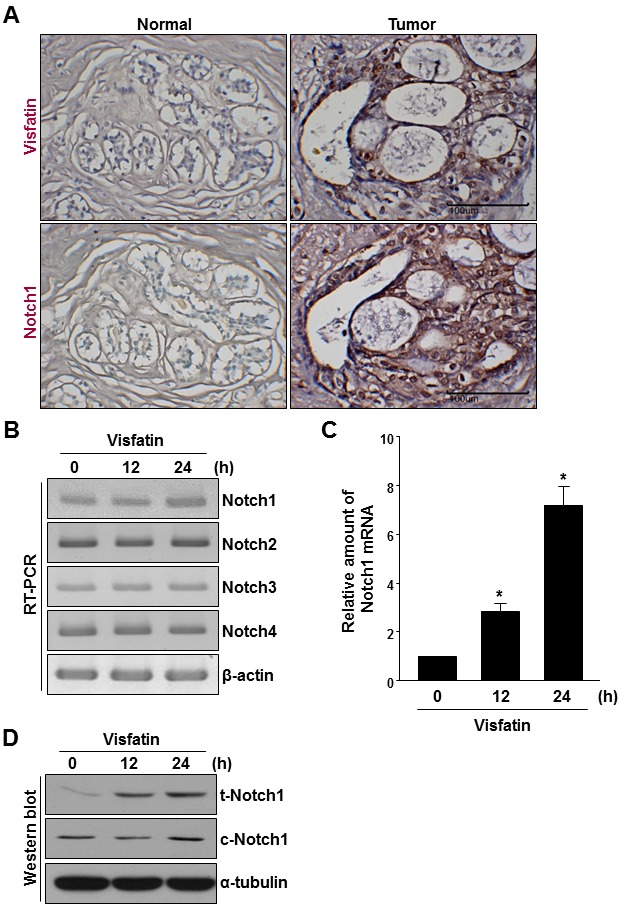
Analysis of visfatin and Notch1 expression in human breast tumor specimens (A) Human breast tumor and normal tissues were immunostained with anti-visfatin and anti-Notch1 antibodies. Each value represents the mean of 30 samples. Scale bar: 100 μm. (B-D) MDA-MB-231 cells were treated with visfatin (500 ng/mL) and polymyxin B (10 μg/mL) for the indicated times. Representative RT-PCR analysis of Notch1, Notch2, Notch3, Notch4, and β-actin mRNA levels (B). Quantitative real-time RT-PCR of Notch1 mRNA levels (C). n=3, * P < 0.01 vs. control. Western blot analysis to detect t-Notch1, c-Notch1, and α-tubulin protein levels (D).

To determine the levels of visfatin and Notch1 proteins in human breast cancer tissues, tissue microarrays containing breast cancer tissue specimens and matched non-tumor tissues were used for immunohistochemical staining of visfatin and Notch1. As shown in Figure 1A, visfatin (12 of 30 cases; 40.0%) and Notch1 (15 of 30 cases; 50.0%) were highly expressed in the malignant epithelium of nearly all human breast cancer tissues, whereas they were not detected in normal breast tissue. Visfatin is known to activate endothelial Notch1 signaling. To examine the role of visfatin in the regulation of Notch1 in breast cancer cells, MDA-MB-231 human breast cancer cells were treated with visfatin for the indicated times and then measured the levels of Notch1 mRNA and protein by qRT-PCR/RT-PCR and western blot analysis, respectively. Visfatin increased the levels of Notch1 mRNA (~7.2-fold), full-length total Notch1 protein (t-Notch1), and cleaved Notch1 protein (c-Notch1) in a time-dependent manner in MDA-MB-231 cells (Figure 1B-D).

### Identification of *Notch1* as a target gene modulated by visfatin in breast cancer cells

**Figure 2:**
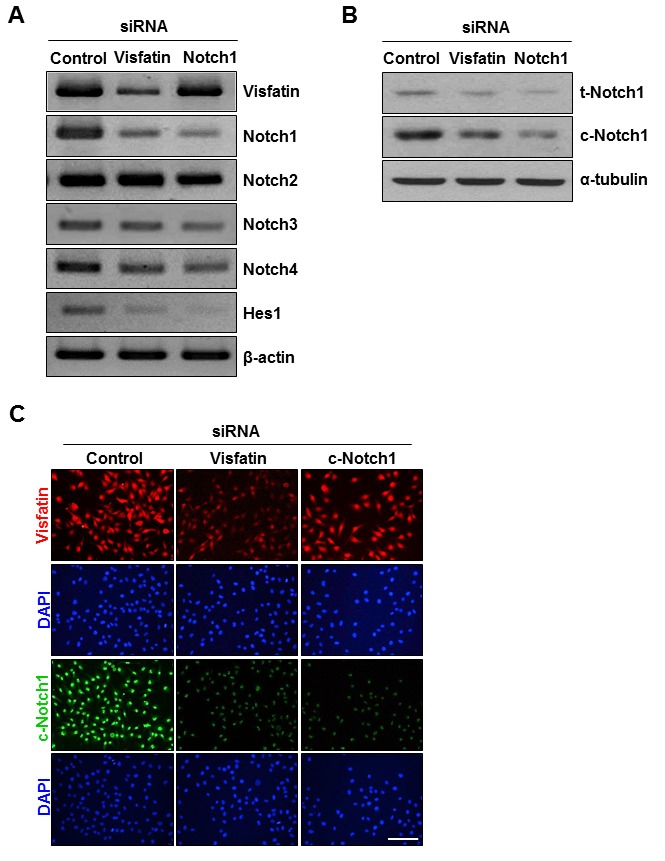
Effects of visfatin depletion on Notch1 expression MDA-MB-231 cells were transiently transfected with control siRNA, visfatin siRNA, or Notch1 siRNA for 48 h. (A) Total RNA was isolated and analyzed by RT-PCR using primers specific to human visfatin, Notch1, Notch2, Notch3, Notch4, and Hes1 transcripts. β-Actin was used as the internal control. (B) The expression of t-Notch1 and c-Notch1 was examined by western blotting using specific antibodies. α-Tubulin was used as the loading control. (C) Immunocytochemical analysis of visfatin (red) and c-Notch1 (green) in MDA-MB-231 cells. The cells were evaluated by a fluorescence microscope. DAPI (blue) stains nuclear DNA. Scale bar: 50 μm.

To further evaluate the effect of visfatin on the *Notch1* gene induction, we used siRNA to knock down visfatin expression. RT-PCR assays and western blot analysis showed reductions in visfatin mRNA and protein levels in visfatin siRNA-transfected cells (Figure 2A, Supplemental Figure 1, and Supplemental Figure 2A). Because the extent of visfatin depletion was greater in cells transfected with siRNA #1 than in cells transfected with siRNA #2 or with a pool of siRNAs (#1 and #2), we used siRNA #1 for the subsequent experiments (Supplemental Figure 1). We examined whether visfatin depletion affected the expression of Notch receptors in breast cancer cells. Among the four Notch receptors, Notch1 was most affected in visfatin-silenced MDA-MB-231 cells (Figure 2A and Supplemental Figure 2B–E). Notch1 mRNA and protein levels were significantly reduced by depletion of visfatin (Figure 2A and B and Supplemental Figure 2F and G). The effect of visfatin depletion on Notch1 protein levels was confirmed by immunocytochemistry (Figure 2C). We subsequently examined whether the repression of Notch1 induced by visfatin depletion was accompanied by the downregulation of *Hes1*, a well-known target gene of Notch1 signaling. The levels of endogenous Hes1 mRNA decreased in accordance with the reduced expression of visfatin and Notch1 (Figure 2A and Supplemental Figure 2H). To investigate whether the effect of visfatin on Notch1 upregulation was dependent on the breast cancer cell type, we knocked down visfatin and Notch1 in other breast cancer cell lines, including BT549, MCF-7, and T47D. The knockdown efficiency was evaluated by RT-PCR. As shown in Supplemental Figure 3, visfatin depletion caused a reduction in Notch1 mRNA level in MDA-MB-231 and BT459 cells, whereas Notch1 mRNA levels were unaffected by visfatin depletion in MCF-7 and T47D cells. Because the extent of Notch1down-regulation by visfatin depletion was higher in MDA-MB-231 than in BT549, we chose MDAMB-231 cells for the following studies.

**Figure 3:**
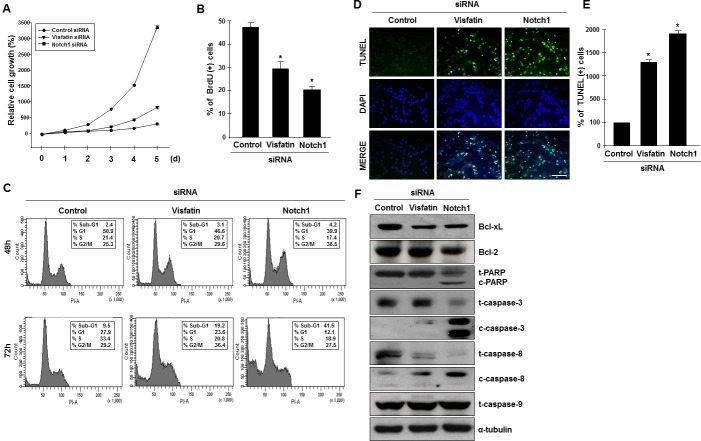
Effects of visfatin or Notch1 depletion on cell proliferation and apoptosis MDA-MB-231 cells were transiently transfected with control siRNA, visfatin siRNA, or Notch1 siRNA. (A) MDA-MB-231 cells were counted at the indicated times after transfection. (B) The BrdU incorporation assay was performed 72 h after transfection. DNA synthesis rates were measured by determining the percentage of BrdU-positive cells with FACS analysis. (C) For cell cycle analysis by flow cytometry, nuclei were stained with propidium iodide to measure the DNA content. (D) After 72 h of transfection, cellular apoptosis was examined by the TUNEL assay. Scale bar: 50 μm. (E) The stained cells (green) were counted, and the percentage of positive cells was calculated. DAPI (blue) stains nuclear DNA. * P < 0.05 vs. control siRNA, n = 3. (F) Western blots were probed with Bcl-xL, Bcl-2, PARP, caspase-3, caspase-8, and caspase-9 antibodies. α-Tubulin served as the loading control.

### Depletion of visfatin and Notch1 decreases cell proliferation and induces apoptosis

We next examined the effects of visfatin and Notch1 depletion on cell growth. An apparent suppression of cell proliferation was observed in both visfatin-depleted and Notch1-depleted MDA-MB-231 cells (Figure 3A and Supplemental Figure 4). To elucidate the potential mechanisms whereby depletion of visfatin or Notch1 inhibits cell proliferation, we performed BrdU incorporation assays. Depletion of visfatin or Notch1 repressed DNA synthesis (Figure 3B). Next, we performed fluorescence-activated cell sorting (FACS) analysis. As shown in Figure 3C, visfatin-depleted cells arrested in G2/M, with sub-G1 (apoptotic) populations present 72 h after transfection. In comparison, among Notch1-depleted cells, a significant increase in the G2/M phase population was observed 48 h after transfection, with a concomitant decrease in the percentage of cells in S phase, when compared to control cells or visfatin-depleted cells. A substantial accumulation of cells in sub-G1 phase was detected after 72 h of transfection with Notch1 siRNA. These results suggested that the cell growth inhibition induced by depletion of visfatin and Notch1 was related to apoptosis. To confirm that visfatin and Notch1 depletion induced apoptosis in MDA-MB-231 cells, we performed TUNEL assays. The number of TUNEL-positive apoptotic cells increased when cells were treated with siRNAs against visfatin and Notch1 (Figure 3D and E). Next, we used western blot analysis to verify apoptosis at the molecular level. Notch1 depletion led to a dramatic reduction in the levels of Bcl-xL and Bcl2, whereas only a reduction in Bcl-xL was detected in visfatin-depleted cells (Figure 3F and Supplemental Figure 5A and B). PARP, caspase-3, and caspase-8 were activated by either visfatin or Notch1 depletion, whereas no change was detected in the levels of caspase-9 (Figure 3F and Supplemental Figure 5C–I).

**Figure 4:**
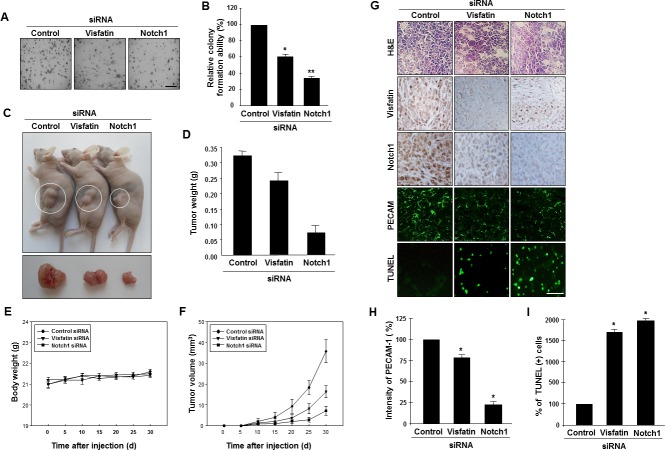
Effects of visfatin or Notch1 depletion on cell and tumor growth (A and B) The effects of visfatin or Notch1 silencing on the colony-forming ability of MDA-MB-231 cells were determined with the soft agar colony formation assay. After 14 days of transfection, photographs were taken (A), and colonies were counted (B). Scale bar: 50 μm. * P < 0.05 vs. control; ** P < 0.01 vs. control siRNA, n = 3. (C–I) MDA-MB-231 cells transfected with control siRNA, visfatin siRNA, or Notch1 siRNA were subcutaneously injected in the flank of athymic nude mice. (C) Mice were sacrificed 30 days after the first injections, and photographs were taken. Images of representative animals with solid tumor and tumor masses are shown. The tumor weight (D) and body weight (E) of mice were measured. (F) Tumor size was determined by its average diameter, which was calculated as (longest length + shortest length)/2. (G) The tumor tissue was removed from mice at 30 days and embedded in paraffin. Tissue sections from xenograft tumor were analyzed by immunohistochemistry using the anti-visfatin, anti-Notch1, and anti-PECAM-1 antibodies. Scale bar: 50 μm. (H) The intensity of PECAM-1 expression was analyzed using the ImageJ program. (I) Xenograft tissue apoptosis was examined by the TUNEL assay and quantified. Each value represents the mean of at least 3 animals, and similar results were obtained in 3 different experiments, * P < 0.05 vs. mice injected with control siRNA.

**Figure 5:**
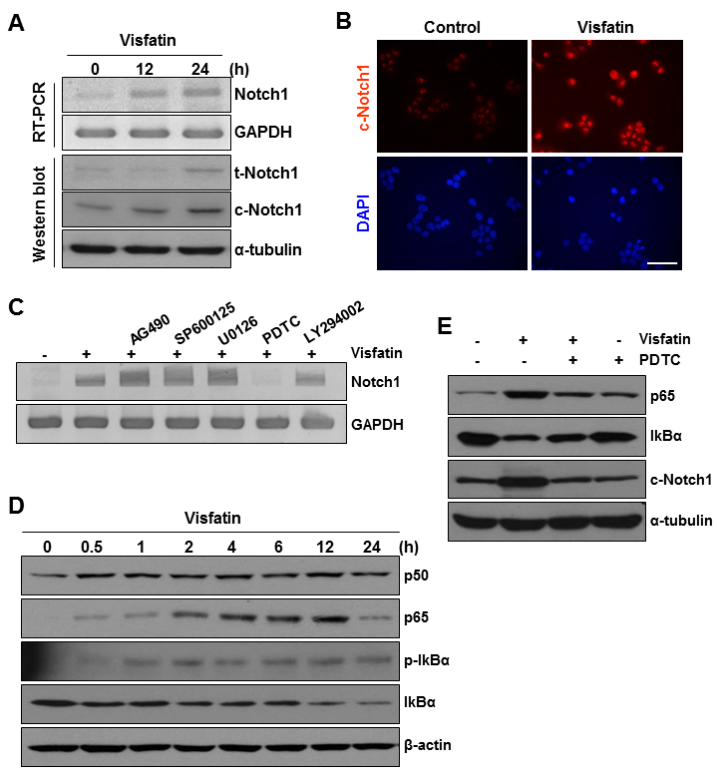
Effects of NF-κB signaling inhibition on visfatin-induced Notch1 upregulation and colony formation of breast cancer cells MCF10A cells were treated with visfatin (500 ng/mL) and polymyxin B (10 μg/mL) for the indicated times. (A) The mRNA levels of human Notch1 and GAPDH were detected by RT-PCR analysis. The expression of t-Notch1 and c-Notch1 was examined by western blotting using specific antibodies. GAPDH and α-tubulin were used as loading controls. (B) Photographs showing cells stained with c-Notch1 antibody (red). The cells were viewed under a fluorescence microscope. Nuclei were counterstained with DAPI (blue). (C) MCF10A cells were pretreated with pharmacological inhibitors such as AG490 (JAK/STAT), SP600125 (JNK), U0126 (MEK1/2), PDTC (NF-κB), and LY294002 (PI3K) for 1 h before exposure to visfatin (500 ng/mL). After 24 h of visfatin treatment, Notch1 mRNA levels were examined by RT-PCR. (D) MCF10A cells were treated with visfatin and polymyxin B for the indicated times. Cell lysates were subjected to western blotting for p50, p65, phospho-/total-IκBα, and β-actin. (E) MCF10A cells were pretreated with PDTC for 1 h before exposure to visfatin (500 ng/mL). After 24 h of visfatin treatment, p65, IκBα, c-Notch1, and α-tubulin protein levels were determined by western blot analysis using specific antibodies for each protein. (F) MDA-MB-231 cells were plated on the surface of soft agar and pretreated with PDTC before exposure to visfatin and polymyxin B. Colony formation was observed, and photographs were taken after 14 days. (G) MDA-MB-231 cells were transiently transfected with control siRNA or Notch1 siRNA. After 48 h of transfection, cells were plated onto the surface of soft agar and treated with visfatin and polymyxin B. Colony formation was observed, and photographs were taken after 14 days. n = 3.

### Depletion of visfatin or Notch1 suppresses cell and tumor growth

To determine whether the visfatin-Notch1 axis plays a role in tumor growth, we used the soft agar assay. MDA-MB-231 cells transfected with visfatin or Notch1 siRNA were assayed for anchorage-independent growth for 14 days. Depletion of visfatin or Notch1 reduced the ability of tumor cells to form colonies in soft agar (Figure 4A and B). Next, we used a xenograft mouse model with MDA-MB-231 cells. Athymic nude mice were injected with cells transfected with visfatin or Notch1 siRNA, and tumor formation was measured after 30 days. A remarkable reduction in tumor weight and volume but not in body weight was observed in mice injected with visfatin or Notch1 siRNA-transfected cells (Figure 4C–F). Tumors were collected for histological analysis, and hematoxylin and eosin staining showed that the tumors formed by the visfatin or Notch1 siRNA-transfected cells had a lower cell density than control siRNA tumors (Figure 4G). A reduction in the levels of visfatin and Notch1 proteins was observed in the tumors derived from the visfatin siRNA-transfected cells (Figure 4G). Similar results were observed in the Notch1 siRNA-transfected group (Figure 4G). In addition, the density of tumor microvessels (PECAM+) in the visfatin siRNA-transfected and Notch1 siRNA-transfected groups was dramatically lower than that in the control group (Figure 4G and H). Moreover, increased numbers of apoptotic cells in the tumors derived from the visfatin and Notch1 siRNA transfectants were observed in the TUNEL assay (Figures 4G and I).

### The NF-κB signaling pathway mediates visfatin-induced Notch1 upregulation

To confirm the direct involvement of visfatin in the regulation of *Notch1* gene expression, we treated non-tumorigenic (MCF10A) and tumorigenic (MDA-MB-231) breast epithelial cells with visfatin for the indicated times and then measured Notch1 mRNA and protein levels by RT-PCR and western blot analysis, respectively. Visfatin treatment increased the levels of Notch1 mRNA, t-Notch1 protein, and c-Notch1 protein in a time-dependent manner in both MDA-MB-231 (Figure 1B and C) and MCF10A (Figure 5A and B) cells. Because the extent of Notch1 induction by visfatin was greater in MCF10A cells than in MDA-MB-231 cells, possibly a result of the low basal level of Notch1 in MCF10A cells, we used MCF10A cells to investigate the underlying molecular mechanisms of visfatin-induced Notch1 upregulation. MCF10A cells were pretreated with the following inhibitors before exposure to visfatin: AG490 (JAK/STAT inhibitor), SP600125 (JNK inhibitor), U0126 (ERK1/2 inhibitor), PDTC (NF-κB inhibitor), and LY294002 (PI3K inhibitor). After pretreating MCF10A cells with these inhibitors, we stimulated the cells with visfatin and evaluated Notch1 induction. As shown in Figure 5C, only the NF-κB inhibitor PDTC significantly suppressed visfatin-induced Notch1 upregulation, suggesting the involvement of the NF-κB pathway in Notch1 induction by visfatin. On the basis of these results, we next analyzed p65 and p50 protein levels in visfatin-treated MCF10A cells and the effects of visfatin on IκBα phosphorylation and degradation by western blot analysis. As shown in Figure 5D, visfatin increased p65 protein levels, which peaked 12 h after visfatin treatment. In contrast, p50 protein levels were almost unchanged. In addition, the pattern of p65 induction in response to visfatin treatment followed the phosphorylation of Ser-32 in IκBα and the degradation of IκBα protein (Figure 5D). We next investigated whether PDTC affected the upregulation of Notch1 by visfatin in MCF10A cells. As shown in Figure 5E, the visfatin-induced increase in Notch1 protein levels in MCF10A cells was attenuated by pretreatment with PDTC, with a concomitant decrease and increase in p65 and IκBα protein levels, respectively, indicating that NF-κB is the major determinant of visfatin-induced Notch1 upregulation.

### The NF-κB-Notch1 signaling pathway mediates visfatin-induced proliferation of breast cancer cells

**Figure 6:**
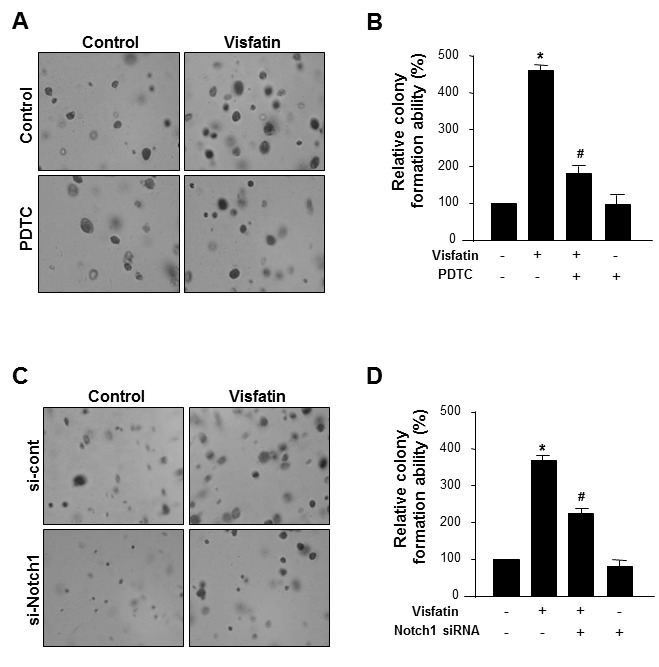
Effect of NF-κB inhibition or Notch1 silencing on the visfatin-induced breast cancer cell proliferation (A-C) MDA-MB-231 cells were plated onto the surface of soft agar and pretreated with PDTC before exposure to visfatin (500 ng/mL) and polymyxin B (10 μg/mL). After 14 days, photographs were taken (A), and colonies were counted (B). (C-D) MDA-MB-231 cells were transiently transfected with control siRNA or Notch1 siRNA. After 48 h of transfection, cells were plated onto the surface of soft agar and treated with visfatin and polymyxin B. After 14 days, photographs were taken (C), colonies were counted (D). Each column represents the mean value of triplicate experiments in each group. * P < 0.05 vs. control, # P < 0.05 vs. visfatin, n = 3.

We further examined whether NF-κB and Notch1 mediated the stimulatory effects of visfatin on breast cancer cells. MDA-MB-231 cells pretreated with PDTC before exposure to visfatin or transfected with Notch1 siRNA in the presence or absence of visfatin were assayed for anchorage-independent growth for 14 days. Blockade of NF-κB signaling (Figure 6A and B) or depletion of Notch1 (Figure 6C and D) markedly reduced the ability of visfatin to proliferate in soft agar. These results suggest that the NF-κB-Notch1 signaling pathway mediates visfatin-induced proliferation of breast cancer cells.

### Clinical correlations between p65 and Notch1 expression in human breast tumor samples

**Figure 7:**
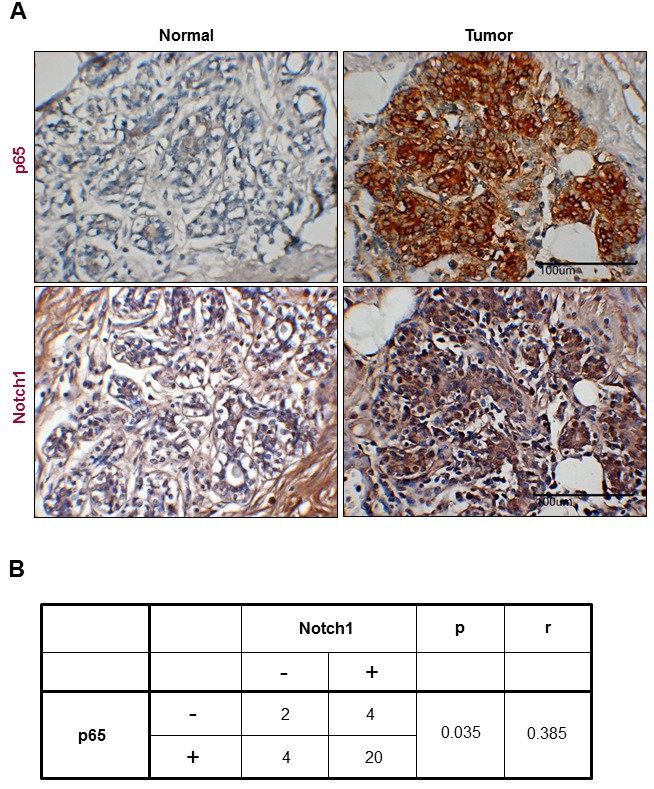
Representative images showing coexpression of p65 and Notch1 in human breast cancer (A) Human breast cancer and normal tissues were immunostained with p65 and Notch1 antibodies, respectively. Each value represents the mean of 30 samples. All tissues are shown at a magnification of 400×. (B) The table presents the correlation between p65 and Notch1 expression in breast cancer.

Because NF-κB p65 mediated visfatin-induced Notch1 upregulation, we investigated whether a correlation between NF-κB p65 and Notch1 expression existed in human breast cancer tissues. The expression of p65 and Notch1 in human breast cancer tissues was assessed by immunohistochemistrical analysis. As shown in Figure 7A, p65 (21 of 30 cases; 70.0%) and Notch1 (15 of 30 cases; 50.0%) were expressed at higher levels in cancer tissues than in non-cancer tissues. Interestingly, Notch1 expression was positively associated with p65 expression, indicating a correlation between p65 and Notch1 in breast tumors (Figure 7B).

## DISCUSSION

Among the Notch receptors with potential oncogenic roles in breast carcinogenesis, Notch1 is the best studied. Overexpression of active Notch1 enhances murine mammary tumor formation [25]. In addition, elevated expression of Notch1 in human breast tumors correlates with poor overall patient survival [26-28], whereas Notch1 overexpression or depletion affects the proliferation, migration, and invasion of breast cancer cells [28-32]. Thus, Notch1 could be a prognostic biomarker for the pathogenesis of breast cancer; as such, it may represent a novel therapeutic target. However, relatively little is known about the upstream modulators and/or molecular mechanisms that regulate Notch1 expression in human breast cancer cells. Here, we provide evidence for the regulation of Notch1 gene expression by visfatin and describe the role of the visfatin-Notch1 axis in breast cancer cells.

Increased levels of visfatin have been implicated in the development of breast cancer [18, 19, 33], and visfatin has been linked to the proliferation and invasion of breast cancer cells. For instance, visfatin enhanced the invasion of MDA-MB-231 human breast cancer cells, whereas visfatin knockdown by siRNA reduced cell invasion [17]. In addition, visfatin increased the proliferation of MCF-7 human breast cancer cells by stimulating cell cycle progression [20]. In the present study, we observed visfatin-dependent regulation of Notch1 gene expression in MDA-MB-231 cells, which affected breast cancer cell growth *in vitro* and *in vivo*. The presence of a visfatin-Notch1 axis is supported by our previous findings in endothelial cells, which showed that visfatin activates Notch1 signaling, leading to endothelial angiogenesis [12]. Considering that Notch1 acts as an oncogene and as a tumor suppressor in human cancer, it will be interesting to investigate whether visfatin also promotes Notch1 expression in other types of cancer, including skin cancer, hepatocellular carcinoma, and head and neck squamous cell carcinoma, in which Notch1 functions as a tumor suppressor.

To date, several signaling pathways, including PI3K [34], p38 MAPK [35], ERK1/2 [11], JNK [36], STAT3 [37], and NF-κB [10], have been reported to contribute to the visfatin-induced activation of downstream target gene transcription. In the present study, we found that visfatin increased the levels of NF-κB p65 as well as Notch1. The induction of Notch1 by visfatin was suppressed by inhibition of the NF-κB signaling pathway, indicating a role for NF-κB p65 as a positive regulator of Notch1 induction by visfatin in breast cancer cells. Emerging evidence supports the existence of functional crosstalk between the NF-κB p65 and Notch1 signaling pathways. For instance, Notch1 signaling can modulate NF-κB-dependent gene transcription by increasing NF-κB expression or activity [38-40]. Conversely, NF-κB p65 induces the expression of the Notch ligand Jagged1, thereby triggering Notch signaling in adjacent cells [41]. In addition, NF-κB p65 enhances the Notch-mediated activation of the *Hes1* promoter by inducing the cytoplasmic retention of the nuclear corepressor N-CoR [42]. At present, we do not know whether NF-κB p65 directly or indirectly mediates the induction of Notch1 expression by visfatin. Thus, future studies will investigate the mechanism underlying the transcriptional activation of Notch1 by NF-κB p65.

In this study, growth inhibition and apoptosis in MDA-MB-231 cells were more sensitive to Notch1 depletion than to visfatin depletion. The Notch1 signaling pathway plays a role in the regulation of mammary tumor stem cell self-renewal and survival [43, 44]. Thus, Notch1 depletion may have had a stronger effect than visfatin depletion because the former targeted the proliferation and survival of the cancer stem cell population as well as the bulk cancer cell population.

Patients with triple-negative breast cancers (TNBC; estrogen receptor-, progesterone receptor-, and HER2-negative) typically have a poor prognosis; thus, there is an increasing need for new therapeutic approaches that target TNBC [45]. Emerging evidence suggests that Notch1 is a biomarker for TNBC [46, 47]. In this report, of four breast cancer cell lines studied, visfatin regulated Notch1 expression only in the MDA-MB-231 and BT-549 cell lines, which are TNBC cell lines, suggesting that targeting the visfatin-Notch1 axis may be an efficient strategy to improve the survival rate of TNBC patients.

In summary, we have identified a pathway that connects visfatin signaling to Notch1 upregulation through NF-κB and shown that downregulation of the visfatin-NF-κB-Notch1 axis inhibits the survival and proliferation of breast cancer cells. Additional *in vivo* studies will be needed to speculate as to whether the visfatin-NF-κB-Notch1 axis has the potential to be a novel target for the treatment of breast cancer and other human cancers.

## MATERIALS AND METHODS

### Reagents and antibodies

Recombinant human visfatin was prepared in our laboratory as previously described.^11^ The endotoxin level in the recombinant visfatin was below 0.05 EU (<5 pg/mL), as determined by Limulus amebocyte lysate testing. Polymyxin B, an endotoxin inhibitor, and pyrrolidinedithiocarbamate (PDTC), an inhibitor of nuclear factor-kappa-B (NF-κB), were obtained from Sigma-Aldrich (St Louis, MO, USA). U0126, an inhibitor of mitogen-activated protein kinase/extracellular signal-regulated kinase 1/2 (MEK1/2), was purchased from A.G. Scientific (San Diego, CA, USA). AG490, an inhibitor of Janus kinase/signal transducers and activators of transcription (JAK/STAT), and DAPT, an inhibitor of Notch, were from Calbiochem (Billerica, MA, USA). SP600125, an inhibitor of c-Jun N-terminal kinase (JNK), was from Enzo Lifesciences (Farmingdale, NY, USA). LY294002, an inhibitor of phosphoinositide 3-kinase (PI3K), was from Promega (Madison, WI, USA). Human anti-visfatin and anti-Notch1 antibodies were purchased from Adipogen (Epalinges, Switzerland) and Abcam (Cambridge, MA, USA), respectively. Antibodies for Bcl-xL, Bcl-2, cleaved caspase-8, cleaved caspase-9, cleaved poly(ADP)-ribose polymerase (PARP), and p50 were purchased from Santa Cruz Biotechnology (Dallas, TX, USA). Rabbit polyclonal anti-phospho-inhibitor of NF-κB (IκB) α (Ser32), anti-cleaved caspase-3, and anti-p65 antibodies, along with mouse monoclonal anti-IκBα antibodies, were obtained from Cell Signaling (Boston, MA, USA). Human CD31 (platelet endothelial cell adhesion molecule (PECAM)-1) antibody was purchased from BD Bioscience (San Jose, CA, USA). Human-specific anti-α-tubulin and anti-β-actin antibodies were purchased from BioGenex (Fremont, CA, USA) and Abcam, respectively. Horseradish peroxidase-conjugated goat anti-rabbit and anti-mouse IgG were purchased from Thermo Fisher Scientific (Waltham, MA, USA). Alexa Fluor® 488-conjugated goat anti-mouse IgG, Alexa Fluor® 488-conjugated goat anti-rabbit IgG, Alexa Fluor® 594-conjugated goat anti-mouse IgG, and Alexa Fluor® 594-conjugated goat anti-rabbit IgG were purchased from Life Technologies (Carlsbad, CA, USA).

### Cell culture

MDA-MB-231, MCF-7, and T47D cells (ATCC; American Type Culture Collection, Manassas, VA, USA) were cultured in Dulbecco's modified Eagle's medium (DMEM; GibcoBRL, Grand Island, NY, USA) containing 10% heat-inactivated fetal bovine serum (GibcoBRL) and 1% penicillin streptomycin (P.S; GibcoBRL) at 37°C in a humidified atmosphere containing 95% air and 5% CO_2_. BT549 cells (ATCC) were cultured in Roswell Park Memorial Institute medium (RPMI; GibcoBRL) containing 10% FBS and 1% P.S at 37°C in a humidified atmosphere containing 95% air and 5% CO_2_. MCF-10A cells (ATCC) were cultured in DMEM/F12 (GibcoBRL) containing 5% horse serum (GibcoBRL), 20 ng/ml EGF (R&D systems, Minneapolis, MN, USA), 0.5 mg/ml hydrocortisone (Sigma-Aldrich), 100 ng/ml cholera toxin (Sigma-Aldrich), 10 mg/ml insulin (R&D systems) and penicillin/streptomycin (GibcoBRL) at 37°C in a humidified atmosphere containing 95% air and 5% CO_2_.

### Transient transfection of small interfering RNA (siRNA)

We designed and synthesized double-stranded siRNA oligonucleotides against visfatin (5′-CCACCCAACACAAGCAAAGUUUAUUTT-3′ and 3′-AAUAAACUUUGCUUGUGUUGGGUGG-5′) and Notch1 (5′-GAACGGGGCUAACAAAGAUTT-3′ and 5′-AUCUUUGUUAGCCCCGUUCTT-3′). Negative-control siRNA was purchased from Bioneer. Oligofectamine (Invitrogen) was used as the transfection reagent according to the manufacturer's recommendations. Cells in 60-mm dishes were transfected at 30% confluence with 200 nM siRNA and 8 μL Oligofectamine for 4 h in minimal serum-free medium without antibiotics. Thereafter, growth medium containing three times the normal concentration of serum was added without removing the transfection mixture, and cells were allowed to grow for an additional 44 h until confluent.

### Reverse transcription polymerase chain reaction (RT-PCR) and quantitative real-time RT-PCR (qRT-PCR)

Total RNA was isolated from MDA-MB-231 cells with a TRIzol reagent kit (Life Technologies). cDNA synthesis was performed using 2 μg of total RNA with a reverse transcription kit (Promega). One microgram of RNA was mixed with random primers and reverse transcribed according to the first-strand method (Invitrogen). cDNA obtained was amplified by PCR. The amplified PCR products were separated on a 1.5% (wt/vol) agarose gel and stained with ethidium bromide. Band intensities were quantified using MetaMorph software. Real-time PCR quantification (qRT-PCR) was performed using a SYBR Green approach (Light Cycler; Roche Applied Science, Penzberg, Germany). Cycling parameters consisted of 1 cycle of 95 °C for 10 min, followed by amplification for 30 cycles of 95 °C for 10 s, 57 °C for 5 s, and 72 °C for 7 s. Subsequently, a melting curve program was applied with continuous fluorescence measurement. The entire cycling process including data analysis was monitored using the LightCycler® software program (version 4.0). β-actin served as an internal control. The relative ratio in the control is arbitrarily presented as 1. Fold changes of target gene mRNA, as determined by real time PCR, are means ± SD from three independent experiments.

### Primer sequences for PCR

The oligonucleotide primers for PCR were as follows: β-actin, 5′-GACTACCTCATGAAGATC-3′ and 5′-GATCCACATCTGCTGGAA-3′; visfatin, 5′-GGATCCATGAATCCTGCGGCAGAAGC-3′ and 5′-CTCGAGATGATGTGCTGCTTCCAGTTC-3′; Notch1, 5′-GCAACAGCTCCTTCCACTTC-3′ and 5′-GCCTCAGACACTTTGAAGCC-3′; Notch1 (for qRT-PCR), 5′- GTCAACGCCGTAGATGACC-3′ and 5′- TTGTTAGCCCCGTTCTTCAG -3′; Notch2, 5′-CCCAATGGGCAAGAAGTCTA-3′ and 5′-CACAATGTGGTGGTGGGATA-3′; Notch3, 5′-TCTTGCTGCTGGTCATTCTC-3′ and 5′-TGCCTCATCCTCTTCAGTTG-3′; Notch4, 5′-TATTCTCATTGCCGGAGCCTCTCGGGAGTA-3′ and 5′-ACCCTCTCCTCCTTGGTTTATGGGCATTTC-3′; Hes1, 5′-AGCACAGAAAGTCATCAAAGCC-3′ and 5′-TTCATGCACTCGCTGAAGCC-3′.

### Western blot analysis

Harvested cells were lysed in a buffer containing 40 mM Tris-Cl, 10 mM EDTA, 120 mM NaCl, 0.1% Nonidet P-40, and a protease inhibitor cocktail (Sigma-Aldrich). Samples containing an equal amount of protein (30 μg/lane) were separated by SDS-PAGE and transferred to a nitrocellulose membrane (GE Healthcare Life Sciences, Pittsburgh, PA, USA). The membrane was blocked with 5% skim milk in PBS or TBS containing 0.1% Tween 20 for 1 h at room temperature and probed with the appropriate antibodies. The signal was developed using an enhanced chemiluminescence detection system (GE Healthcare Life Sciences).

### Immunocytochemistry

Cells cultured on a cover glass were fixed in 4% paraformaldehyde for 10 min, blocked with 0.5% Triton X-100/PBS for 5 min, and then reacted with the appropriate primary antibodies and Alexa Fluor® 488- and 594-conjugated secondary antibodies. Coverslips were mounted in Vectastain containing 4′,6-diamidino-2-phenylindole (DAPI; Vector Laboratories, Burlingame, CA, USA). Cells were analyzed with fluorescence microscopy (Nikon, Gotenba, Shizuoka, Japan).

### BrdU incorporation assay

To evaluate cell proliferation, we used a FITC BrdU Flow kit (BD Pharmingen, Franklin Lakes, NJ, USA) according to the manufacturer's instructions. siRNA-transfected MDA-MB-231 cells were labeled with BrdU for 3 h, washed, and fixed and permeabilized with BD Cytofix/Cytoperm buffer. After repeated rounds of incubation on ice, washing, and centrifugation, cells were treated with DNase for 1 h at 37°C to expose the BrdU epitope. Thereafter, cells were washed, stained with fluorescent anti-BrdU for 20 min at room temperature, washed again, and analyzed using a FACS Calibur cell analyzer (BD Bioscience, Franklin Lakes, NJ, USA).

### Flow cytometry analysis

siRNA-transfected MDA-MB-231 cells (5 × 10^5^ cells) were seeded in 60-mm dishes and cultured at 37°C for different time periods. The cells were washed twice in 1× PBS, fixed in chilled 70% ethanol, stained with 5 μg/mL propidium iodide (Sigma-Aldrich) at room temperature for 10 min, and analyzed using a FACS Calibur cell analyzer (BD Bioscience). The cell cycle profile was determined using the ModFit LT software.

### TUNEL assay

Apoptotic cells were confirmed using the DeadEnd™ Fluorometric TUNEL System (Promega) in accordance with the manufacturer's instructions. siRNA-transfected cells were incubated for 72 h, fixed in 4% paraformaldehyde for 25 min at 4°C, and permeabilized with 0.2% Triton X-100 for 5 min at room temperature. The free 3′ ends of fragmented DNA were enzymatically labeled with the TdT-mediated dUTP nick end labeling (TUNEL) reaction mixture for 60 min at 37°C in a humidified chamber. Labeled DNA fragments were monitored using fluorescence microscopy (Nikon).

### Colony formation assay

siRNA-transfected MDA-MB-231 cells (1 × 10^3^ cells per well) in complete cell culture medium were seeded in 24-well plates in 0.3% soft agar and layered on top of a 0.5% soft agar base layer. Colonies were counted and analyzed after 2 weeks.

### Tumor xenograft model

siRNA-transfected MDA-MB-231 cells (1 × 10^7^ cells) were subcutaneously injected into either side of the flank area of 6-week-old female athymic nude mice. Mice were divided into 2 groups of 5 animals each when the tumor size exceeded 4 mm in diameter. The mice were weighed, and the tumor size was measured using a caliper every Monday and Friday for 24 days. All animal care and experiments were performed in accordance with the Institutional Guidelines of the Animal Care and Use Committees of Pusan National University.

### Immunohistochemistry

Xenograft tissues were prepared, and thin sections (4 μm) from selected areas were used. In brief, after deparaffinization and blocking of endogenous peroxidase activity, antigen retrieval was routinely performed using citrate buffer (pH 6.0) (Invitrogen). The primary antibodies used in this study were anti-visfatin, anti-Notch1, and anti-PECAM-1. To detect these antibodies, Alexa Fluor® 488-conjugated anti-mouse IgG (1:200), Alexa Fluor® 594-conjugated anti-rabbit IgG (1:200), biotin-conjugated anti-mouse IgG (1:200), and biotin-conjugated anti-rabbit IgG (1:200) were used. Isotype controls were stained with one of the secondary antibodies to verify specificity. Immunofluorescence staining was visualized using a Nikon Digital Sight DS-SMc camera attached to a Nikon ECLIPSE 55i microscope. Immunohistochemistry for human breast tumor samples was performed using human breast cancer-tissue microarrays purchased from Super Bio Chips (SuperBioChips Laboratories, Seoul, South Korea). The tumor tissues were obtained from surgical specimens of Korean breast cancer patients. Thin (4 μm) unstained sections were used in immunohistochemical stain. To be brief, after deparaffinization and rehydration with 100% alcohol, endogenous peroxidase activity was inhibited by the treatment of 3% H_2_O_2_ for 20 min at RT. Then, antigen retrieval for p65 was performed by boiling slides for 15 min in citrate buffer (pH 6.0) (Invitrogen). The primary antibodies, such as anti-visfatin, anti-Notch1 and anti-p65, were applied overnight at 4°C. To visualize binding Notch1 and visfatin antibodies, SuperPicture™ 3^rd^ Gen IHC Detection kit (Invitrogen) was used, while CSA II Biotin-free Signal Tyramide Signal Amplification System (Dako, Copenhagen, Denmark) system was used for p65, respectively. After counterstaining with hematoxylin and dehydration, each slide was mounted using malinol. Human oral squamous cell carcinoma tissue sections, which were known to overexpress Notch1, p65, and visfatin proteins, were used as positive controls in every immunohistochemical procedures. As negative control, normal goat serum solution was used instead of the primary antibody solution.

### Evaluation of immunohistochemical stains

The stained slides of tissue microarrays were evaluated and scored by an oral pathologist (MH Ryu), who did not know about the results of other experiments. The expression levels of visfatin, Notch1, and p65 were evaluated via a semi-quantitative method. The level of immunoexpression was determined by multiplying the intensity and the extent of positive stained cells, to produce a value between 0 and 12. The intensity of positive stained epithelial cells was categorized into 4 groups: (a) No staining (score 0); (b) weak staining (score 1); (c) moderate staining (score 2); and (d) strong staining (score 3). The extent of positive stained cells was graded into five easily reproducible groups: (a) No detectable expression (score 0); (b) positive expression in 1-25% of cells (score 1); (c) positive expression in 26-50% of cells (score 2); (d) positive expression in 51-75% of cells (score 3); and (e) positive expression in greater than 75% of cells (score 4). Then, we calculated the mean value of visfatin, Notch1, and p65 immunoexpression respectively, and set expression greater than this mean value to be high, otherwise low.

### Statistical analysis

Data are the mean ± standard deviation (S.D.) obtained from at least 3 independent experiments. Statistical comparisons between groups were performed either by one-way analysis of variance followed by Student's t-test or by Kolmogorov-Smirnov test. Kruskal-Wallis test (Mann-Whitney U test) was performed to analyze the differences between normal breast tissue group and the breast cancer group. In order to evaluate the relationship between the immunoexpression index of proteins, Spearman correlation coefficient was calculated. Statistical significance was considered for *p* <.05. All statistical analyses were performed using Window PASW (Predictive Analytics SoftWare) version 21.0 (SPSS Inc, Chicago, IL, USA).

## SUPPLEMENTARY MATERIAL AND FIGURES


