# Supplementary material for: Visfatin promotes cell and tumor growth by upregulating Notch1 in breast cancer
Source: Oncotarget. 2014 Jun 10;5(13):5087–99. doi: 10.18632/oncotarget.2086 (PMC4148124; doi:10.18632/oncotarget.2086)
Supplement: Supplementary file 1 [file oncotarget-05-5087-s001.pdf]

Visfatin promotes cell and tumor growth by upregulating Notch1 in breast cancer

Supplementary Material

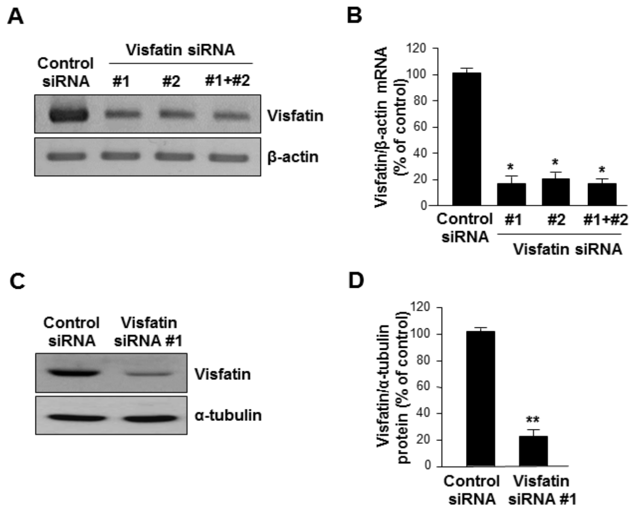

Supplemental Figure 1: Effects of Visfatin siRNA on endogenous Visfatin gene expression.

(A and B) MDA-MB-231 cells were transiently transfected with control siRNA, two different pairs of Visfatin siRNA (#1 and #2), or pooled siRNA (#1 + #2). (A) RT-PCR was performed to analyze the knockdown of endogenous Visfatin gene expression by siRNA. β-Actin was used as an internal control. (B) The graphs show the densitometric analysis of the relative mRNA levels. (C and D) MDA-MB-231 cells were transiently transfected with control siRNA and Visfatin siRNA (#1). (C) Visfatin protein expression was examined by western blotting using specific antibodies, with α-tubulin as the loading control. (D) The graphs show the densitometric analysis of relative protein levels. The density of the control bands (untreated) was set to 100%.

* $P < 0.05$ vs. control; ** $P < 0.01$ vs. control siRNA, n = 3.

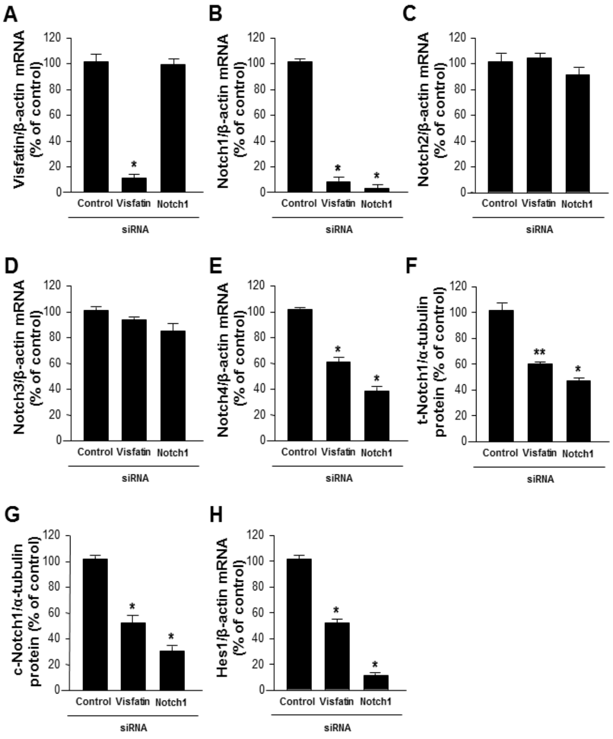

Supplemental Figure 2: Effects of Visfatin or Notch1 depletion on Notch receptors and HES1 expression. MDA-MB-231 cells were transiently transfected with control siRNA, Visfatin siRNA, or Notch1 siRNA. After 48 h of transfection, cell extracts were subjected to RT-PCR (A–F) and western blot analysis (G and H) to detect the mRNA and protein levels of the indicated genes, respectively. The density of the control bands (untreated) was set to 100%. * $P < 0.05$ vs. control; ** $P < 0.01$ vs. control siRNA, $n = 3$.

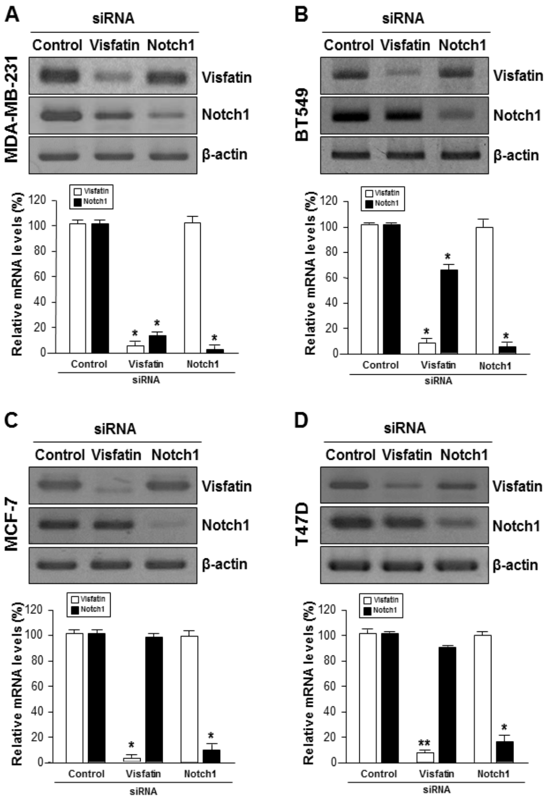

Supplemental Figure 3: Effect of Visfatin depletion on Notch1 gene expression in various types of breast cancer cells. MDA-MB-231 (A), BT549 (B), MCF-7 (C), and T47D (D) cells were transiently transfected with control siRNA, Visfatin siRNA, or Notch1 siRNA. After 48 h of transfection, total RNA was isolated and analyzed by RT-PCR using primers specific to human Visfatin, Notch1, and β-actin. The graphs show the densitometric analysis of relative mRNA levels. The density of the control bands (untreated) was set to 100%. * $P < 0.05$ vs. control; ** $P < 0.01$ vs. control siRNA, n = 3.

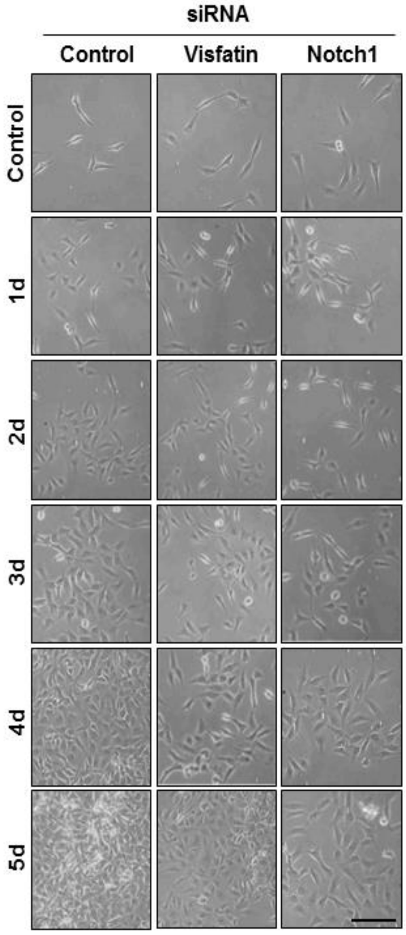

Supplemental Figure 4: Effect of Visfatin or Notch1 depletion on breast cancer cell growth.

MDA-MB-231 cells were transiently transfected with control siRNA, Visfatin siRNA, or Notch1 siRNA. After transfection, cells were plated onto 24-well culture dishes. The cells were checked daily using an inverted microscope, and photographs were taken. Scale bar: 50 μm.

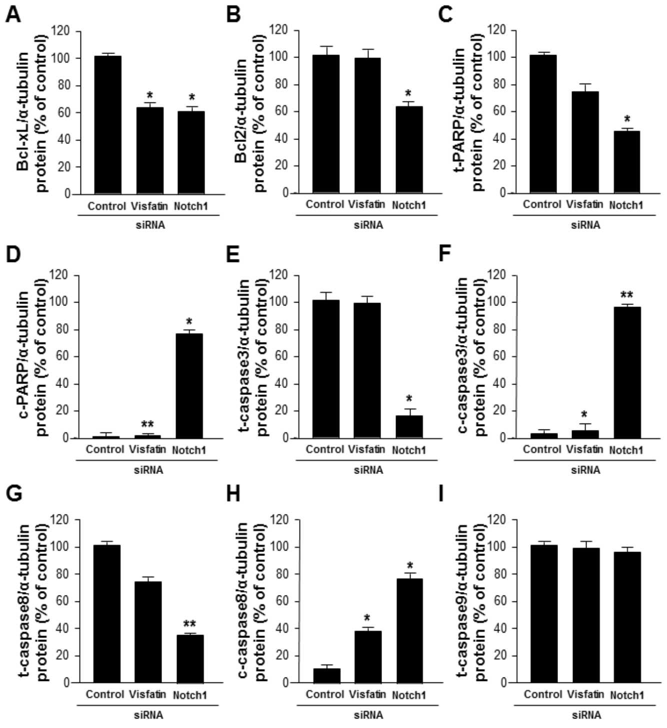

Supplemental Figure 5: Effects of Visfatin or Notch1 depletion on cell cycle- and apoptosis-related gene expression. MDA-MB-231 cells were transiently transfected with control siRNA, Visfatin siRNA, or Notch1 siRNA. After 72 h of transfection, cell extracts were subjected to western blot analysis to detect Bcl-xL (A), Bcl-2 (B), PARP (C and D), caspase-3 (E and F), caspase-8 (G and H), and caspase-9 (I) protein levels. Bands were analyzed by scanning densitometry to measure relative changes in protein expression. * $P < 0.05$ vs. control; ** $P < 0.01$ vs. control siRNA, n = 3.
